# Decreased and Heterogeneous Neutralizing Antibody Responses Against RBD of SARS-CoV-2 Variants After mRNA Vaccination

**DOI:** 10.3389/fimmu.2022.816389

**Published:** 2022-04-06

**Authors:** Pablo Hernández-Luis, Ruth Aguilar, Judit Pelegrin-Pérez, Gemma Ruiz-Olalla, Alberto L. García-Basteiro, Marta Tortajada, Gemma Moncunill, Carlota Dobaño, Ana Angulo, Pablo Engel

**Affiliations:** ^1^ Immunology Unit, Department of Biomedical Sciences, Faculty of Medicine and Health Sciences, University of Barcelona, Barcelona, Spain; ^2^ ISGlobal, Hospital Clínic, University of Barcelona, Barcelona, Spain; ^3^ Centro de Investigacão em Saúde de Manhiça, Maputo, Mozambique; ^4^ Centro de Investigación Biomédica en Red de Enfermedades Infecciosas (CIBERINFECT), Barcelona, Spain; ^5^ Occupational Health Department, Hospital Clínic, University of Barcelona, Barcelona, Spain; ^6^ Institut d’Investigacions Biomèdiques August Pi i Sunyer, Barcelona, Spain

**Keywords:** SARS-CoV-2 variants, neutralizing antibodies (NAB), receptor binding domain (RBD), mRNA vaccines, healthcare workers (HCW), BNT162b2 mRNA, mRNA1273, COVID-19

## Abstract

The rapid spread of Severe Acute Respiratory Syndrome Coronavirus 2 (SARS-CoV-2) emerging variants raises concerns about their capacity to evade immune protection provided by natural infection or vaccination. The receptor-binding domain (RBD) of the viral spike protein is the major target of neutralizing antibodies, and viral variants accumulate mutations in this region. In this study, we determined the antibody neutralization capacity against the RBD of SARS-CoV-2 variants Alpha (B.1.1.7), Gamma (P.1), Epsilon (B.1.427), Kappa (B.1.617.1), and Delta (B.1.617.2) in a cohort of healthcare workers naturally infected or receiving COVID-19 mRNA vaccines from Moderna or Pfizer-BioNTech. We show that the five RBD variants displayed an augmented binding to ACE2 compared to the original Wuhan strain. The most significant increase was observed in variants Epsilon and Delta, containing mutation L452R. Using a flow cytometry cell-based assay, we found that SARS-CoV-2-infected subjects presented low levels of RBD-specific neutralizing antibodies against all variants analyzed, except Alpha. However, the neutralizing activity incremented considerably after a subsequent mRNA-vaccine dose, to levels significantly higher than those in naïve individuals receiving two vaccine doses. Importantly, we observed partially impaired neutralizing responses against most variants in fully vaccinated individuals. Variants Gamma and Kappa encompassing RBD E484K/Q mutations presented the highest neutralizing resistance. Furthermore, a wide heterogeneity in the magnitude of RBD-specific neutralizing responses against all tested SARS-CoV-2 variants following both mRNA vaccines was detected. Altogether, our findings provide important knowledge regarding SARS-CoV-2 vaccine-induced immunity, and should be very useful to guide future vaccination regimens and personalized vaccine approaches.

## Introduction

The severe acute respiratory syndrome coronavirus 2 (SARS-CoV-2), as other RNA viruses, is prone to introduce errors in its genome, and consequently a large number of variants have been identified since the outbreak of the virus in 2019 (1). Some of these variants have rapidly spread and supplanted the original Wuhan strain. SARS-CoV-2 enters host cells *via* the binding of the spike (S) glycoprotein to angiotensin converting enzyme (ACE2) through the S1 receptor-binding domain (RBD), which is highly variable and the primary target of the neutralizing antibody response (2). The elevated frequency of mutations associated with this region could alter the interactions with the host receptor, affecting viral transmissibility by increasing the affinity to ACE2, or promoting the resistance against neutralizing antibodies, thereby jeopardizing the efficacy of vaccines and antibody therapies (3, 4).

Different types of COVID-19 vaccines are under development, and a number of them have become available in the last year and used worldwide. There are five main types of vaccines against SARS-CoV-2: live attenuated and inactivated, vector-based, protein subunit, virus-like particles, and nucleic acid (DNA and RNA) vaccines (5). The two COVID-19 mRNA vaccines, BNT162b2, produced by Pfizer-BioNTech, and mRNA-1273, from Moderna, have been highly effective in preventing symptomatic, particularly severe disease (6). Both vaccines are based on the spike (S) surface glycoprotein of the original SARS-CoV-2 Wuhan strain, and share the same technological approach. However, each of these vaccines uses a somewhat different system for the intracellular mRNA delivery, different total dose of mRNA, and dosing schedule (7, 8). Largely based on vaccine supply and epidemiological status, countries have followed different vaccination policies and strategies, and there is still a need to adjust and optimize the effectiveness of these strategies in the context of vaccine performance among different population groups and dissemination of the new viral variants. Most of the current vaccines are highly effective against the early circulating variants, but their effectiveness against the new emerging variants in different populations needs to be established (6).

Infection and vaccination of naïve people induce both humoral and cellular responses. In fact, there are reports that indicate that a potent SARS-CoV-2 cellular response may be present in individuals without detectable levels of antibodies (9). Although antiviral T cells certainly contribute to protection (10, 11), several studies have shown that vaccine-induced neutralizing antibodies to the RBD of the SARS-CoV-2 S protein are a key defense mechanism and highly predictive of protection (12–14).

Therefore, the goal in this study was to evaluate the RBD-antibody neutralizing capacity against different variants of concern and interest elicited in healthcare workers by the two approved mRNA-based vaccines, the Moderna mRNA-1273 and the Pfizer-BioNTech BNT162b2. Both vaccines had been already administered to millions of people in different parts of the world (8).

## Material And Methods

### Study Population

We have tested 103 samples of healthcare workers from the Hospital Clínic in Barcelona, Spain (15), being 67% female and with a mean (SD) age of 40.6 (10.3) years old. Thirty-six were pre-exposed and unvaccinated; 20 were naïve and vaccinated with two doses of BNT162b2; 27 were vaccinated with one dose of mRNA-1273 (15 pre-exposed and 12 naïve), and 20 were naïve and vaccinated with 2 doses of mRNA-1273 (**Supplementary Table 1**). Previous exposure was defined as evidence of SARS-CoV-2 seropositivity or rRT-PCR positivity. Participants were recruited at the peak of the first wave of the pandemic in Barcelona, Spain, and followed up for one year. Venous or finger prick blood was collected at different visits after recruitment and immunization with one or two doses of the mRNA-1273 (Spykevax) (16) or two doses of the BNT162b2 (Comirnaty) (17), products from Moderna Biotech and Pfizer-BioNTech, respectively. Samples analyzed from the vaccinated participants were collected 10 to 21 days post 1^st^ dose and 10 to 34 days after the 2^nd^ dose. Samples analyzed from the pre-exposed non-vaccinated participants were collected 12 months after recruitment. Ten pre-pandemic samples were also assayed as negative controls. All plasma samples were isolated and cryopreserved at -80°C.

### Production of RBD Variants

Splicing by overlap extension (SOE) PCR was used to progressively introduce mutations into the wild-type (WT, Wuhan strain) RBD-mFc plasmid, encoding the RBD (amino acids 333-529) of the SARS-CoV-2 S protein fused to the Fc region of murine IgG1 in the pFUSE-mIg1-Fc vector (InvivoGen). Primers used for the construction of the RBD-mFc proteins for the different variants (Alpha RBD-mFc, containing mutation N501Y; Gamma RBD-mFc containing mutations K417T, E484K, and N501Y; Delta RBD-mFc containing mutations L452R and T478K); Epsilon RBD-mFc containing mutation L452R; and Kappa RBD-mFc containing mutations L452R and E484Q), were obtained from Sigma-Aldrich and are shown in **Supplementary Table 2**. SARS-CoV-2 RBD sequences were downloaded from the GISAID (18). We acknowledge all authors that submitted sequences to the GISAID, which are listed in (https://www.gisaid.org). All nucleotide changes were confirmed by DNA sequencing. HEK-293T cells were transiently transfected with the different plasmids using 1mg/mL of polyethylenimine (Sigma-Aldrich) in OPTIMEM medium (Gibco) for seven days as previously described (19). Supernatants were collected after centrifugation at 1500 x g, for 10 min, and the RBD-mFc proteins were quantified with an in-house sandwich ELISA, employing 96 well ELISA plates (Costar), goat anti-mouse IgG (Sigma-Aldrich), and goat anti-mouse IgG-HRP anti-sera (Sigma-Aldrich). Purified mouse IgG (Sigma-Aldrich) was used as a standard in the ELISA.

### Neutralization Assay

Neutralizing capacity was measured by a flow cytometry cell-based assay as previously described (20). To this end, we employed a 300.19 pre-B murine cell line stably transfected with the cDNA of the human ACE2 receptor that we specifically generated for this purpose (20). Briefly, the stable cell line expressing ACE2 was incubated with WT or each of the variant RBD-mFc fusion proteins (4μg/mL), previously exposed to the different plasma samples at a dilution 1:50 (non-vaccinated participants) or 1:400 (vaccinated participants). Plasma sample dilutions used reflect the different levels of neutralizing antibodies produced after vaccination as compared to those in naturally exposed individuals. These dilutions were selected based on a previous report of a cohort of healthcare workers that included the study population analyzed here, in which we evaluated the levels of neutralizing antibodies against WT RBD in SARS-CoV-2 exposed non-vaccinated subjects (after one year follow-up) and in individuals receiving the Moderna or Pfizer-BioNTech vaccines (21). Cells were stained with anti-mouse IgG-PE (Jackson ImmunoResearch), washed, and analyzed by flow cytometry using standard procedures. Percentage of neutralization was determined by the reduction of the mean fluorescence intensity (MFI) corresponding to the RBD-ACE2 binding in the presence of each plasma sample relative to the RBD-ACE2 binding in the absence of plasma sample. In figures, dots represent the mean of two independent determinations from each individual serum sample analyzed, and black bars indicate median % of neutralization for each group.

### Statistical Analysis

Statistical analyses were performed using GraphPad Prism 8 and R software, and differences were considered significant if *P*<0.05. A Shapiro-Wilk test was carried out to evaluate the normality of the data. The Friedman test was employed for paired data, to assess overall differences between groups, and the non-parametric Wilcoxon signed-rank and Mann-Whitney tests (based on the non-normal distribution of the data of several groups) were applied to analyze changes in paired and unpaired data, respectively.

## Results And Discussion

**Figure 1:**
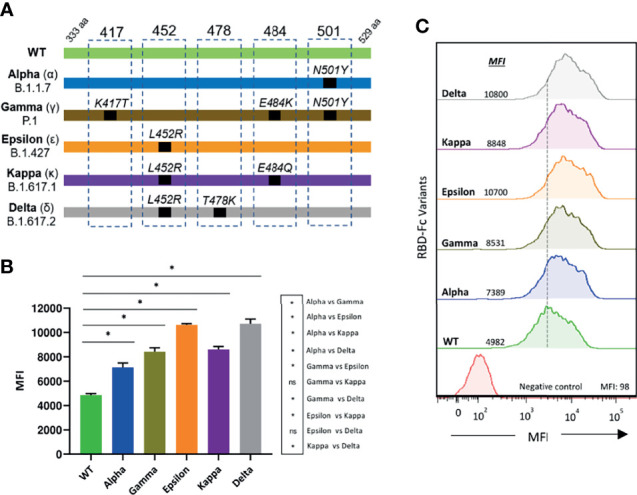
Binding of SARS-CoV-2 RBD variants to ACE2 **(A)** Schematic illustration of the RBD variants evaluated. Residue modifications in comparison to the WT RBD sequence are indicated **(B)** The interaction of the different RBD-Fc proteins to 300.19-ACE2 expressing cells was analyzed by flow cytometry. Measurements were performed in triplicates. Statistical differences were determined by the Mann-Whitney test. Statistical significance: *P < 0.05, ns, non-significant. **(C)** A representative histogram for each variant is shown. MFI, mean fluorescence intensity.

We focused on RBDs of three SARS-CoV-2 variants of concern, the Alpha (B.1.1.7), Gamma (P.1), and Delta (B.1.617.2) variants, first detected in United Kingdom, Brazil, and India, respectively, and of two variants of interest, the Epsilon (B.1.427), and Kappa (B.1.617.1) variants, first identified in Southern California and India, respectively (schematically illustrated in **Figure 1A**). RBD-Fc fusion proteins of the five variants were generated and evaluated by flow cytometry for binding to the ACE2 receptor expressed at the cell surface. All the tested RBD variants showed an augmented interaction with ACE2 as compared to WT RBD. Epsilon and Delta variants presented the highest binding capacity (2.5-fold increase), followed by Gamma and Kappa (2.2-fold increase), and then Alpha (1.5-fold increase) (**Figures 1B, C**). Consistent with previous studies, these results point to a substantial contribution of the L452R mutation to the increased binding affinity of these variants to ACE2 (3, 22).

**Figure 2:**
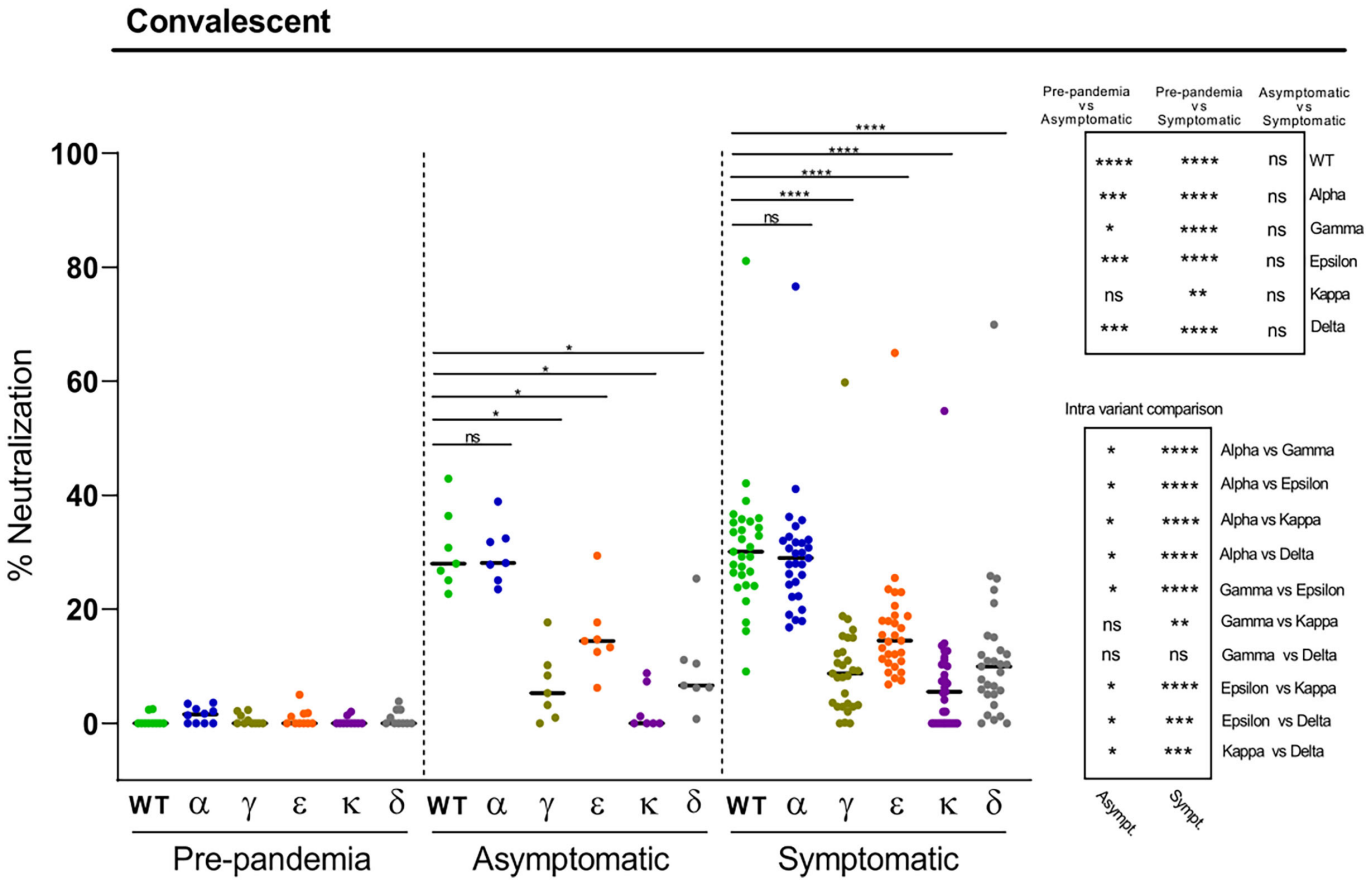
Neutralization of SARS-CoV-2 variants by plasma from convalescent individuals. Comparative neutralizing capacity by plasma from SARS-CoV-2 previously exposed asymptomatic (n=7) and symptomatic (n=29) subjects, and negative pre-pandemic controls (n=10). Plasma dilution used, 1:50. Dots represent the mean of two independent determinations from each individual serum sample analyzed, and black bars indicate median % of neutralization for each group. Statistical differences were determined by Mann-Whitney (for two independent samples) or Wilcoxon (for two relating samples) tests. Statistical significance: *P < 0.05, **P < 0.01, ***P < 0.001, ****P < 0.0001, ns, non-significant. Friedman test *P*-values: pre-pandemic = 0.26, asymptomatic = 2.48·10^-5^, and symptomatic = 1.99·10^-23^.

Next, we evaluated the antibody neutralization capacity against the SARS-CoV-2 variants of plasma from healthcare workers naturally infected with the Wuhan strain, collected one year after the pick of the first wave of the pandemic (February-March 2020). We used a validated cell-based flow cytometry assay previously developed in our laboratory for the WT Wuhan strain that assesses the percentage of inhibition of the binding of RBD-Fc to ACE2-expressing cells (20). A number of pre-pandemic plasma samples were used as controls, which in some cases exhibited minimal levels of neutralization (below 5%), most likely due to their cross-reactivity with other human coronaviruses, as previously described (20). When comparing to the WT strain, all except the Alpha variant, displayed a higher degree of resistance to anti-RBD antibodies present in these subjects (**Figure 2**). The most pronounced decrease on neutralization was observed for the Kappa, followed by the Gamma and Delta variants (**Figure 2**). In fact, a number of the convalescent plasma analyzed fell below the limit of detection of the assay for these three variants. Less pronounced reductions were measured for the Epsilon variant. Interestingly, no differences in plasma neutralization capacity were observed between asymptomatic and symptomatic individuals. Thus, these data show that natural infection with the SARS-CoV-2 Wuhan strain produces low levels of neutralizing antibodies against the new emerging variants, potentially rendering a significant proportion of individuals unprotected from humoral responses. Yet, some of these subjects may be still protected, since individuals without detectable antibody responses have been shown to be able to build potent SARS-CoV-2 T cell responses (9).

**Figure 3:**
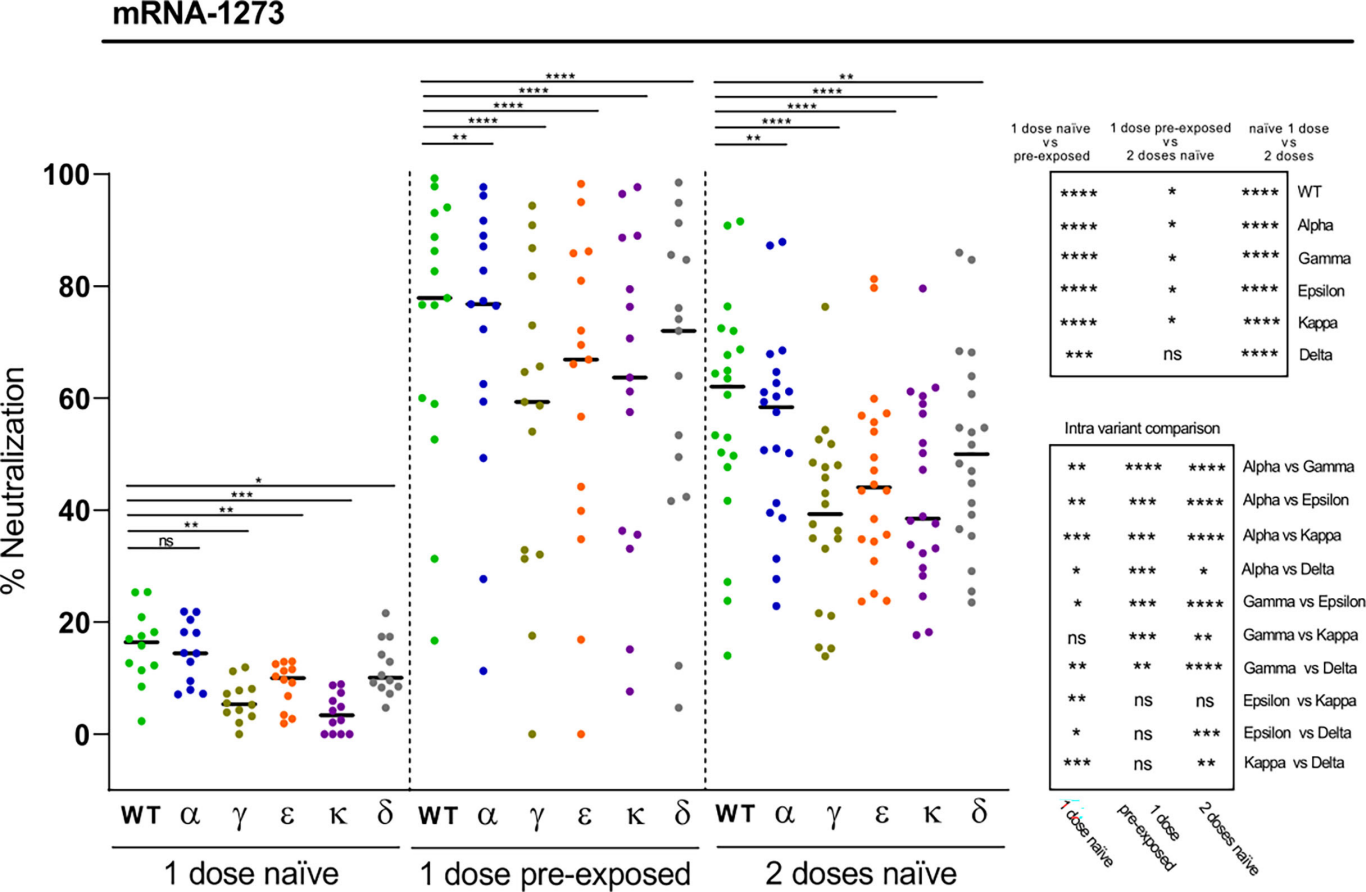
Neutralization of SARS-CoV-2 variants by plasma from mRNA-1273 vaccine recipients. Comparative neutralizing capacity by plasma from naïve (n = 12) and pre-exposed (n = 15) mRNA-1273 recipients of 1 dose, and naïve (n = 20) recipients of 2 doses. Plasma dilution used, 1:400. Dots represent the mean of two independent determinations from each individual serum sample analyzed, and black bars indicate median % of neutralization for each group. Statistical differences were determined by Mann-Whitney (for two independent samples) or Wilcoxon (for two relating samples) tests. Statistical significance: *P < 0.05, **P < 0.01, ***P < 0.001, ****P < 0.0001, ns, non-significant. Friedman test *P*-values: 1 dose naïve = 1.20·10^-6^, 1 dose pre-exposed = 8,85·10^-11^, and 2 doses naïve = 2.06·10^-13^.

We next asked to which extent the Moderna (mRNA-1273) vaccine induced neutralizing antibodies against the different variants depending on previous SARS-CoV-2 exposure or number of doses. The neutralization levels after the first dose in naïve subjects were modest, especially against the Kappa and Gamma variants (**Figure 3**). However, the neutralizing potency substantially increased when naturally infected individuals got one dose of the mRNA-1273 vaccine (**Figure 3**). Most of these individuals reached levels of neutralization that were statistically significantly higher as compared with naïve individuals that received the complete two-dose schedule of the vaccine (**Figure 3**). In these two latter cases, and paralleling the trend observed after natural infection, significant reductions in neutralization as compared to WT RBD were obtained for the Gamma and Kappa variants, followed by the Epsilon and Delta variants (**Figure 3**). A minimal decrease on neutralization was observed for the alpha variant. These data suggest that delaying the second mRNA-1273 dose in SARS-CoV-2 unexposed subjects may not be an adequate strategy as they may not be fully protected and therefore contribute to the spread of emerging variants. Moreover, the results also show that vaccination of convalescent subjects is highly recommended, and suggest that, overall, naturally infected individuals would not require a second dose of the vaccine, since most of them have substantial neutralizing antibody levels against the different variants. However, the heterogeneity of responses observed, with a few individuals presenting modest RBD-specific antibody neutralization capacity against some of the variants, underscores in these cases the need of a complete two-dose vaccination scheme to achieve a more effective protection (**Figure 3**).

We did not observe a correlation between RBD binding efficiency to ACE2 and neutralization resistance, indicating that the immune escape capacity of SARS-CoV-2 variants, such as Gamma and Kappa, may be largely driven by their ability to avoid the neutralization of antibodies induced by the WT Wuhan strain. Of note, these two variants have in common the presence of the E484K/Q mutation, with Kappa presenting in addition the L452R substitution. This later variant has also been reported to evade the neutralization of therapeutic monoclonal antibodies and antibodies triggered by infection and vaccination (23). Thus, as it has been described elsewhere, our findings indicate that the Kappa variant has evolved to enhance viral fitness by simultaneously evading the host neutralizing antibody responses and increasing ACE2 binding affinity (3). Our data also support that the RBDs of the Epsilon and Delta variants, which contain the L452R substitution, augment infectivity largely as a result of their improved affinity to the host receptor (24). Finally, the greater transmissibility of the Delta variant, with a rapid spread during the summer 2021 becoming the dominant strain in Europe at that time, may be also mainly due to its enhanced RBD receptor affinity (22, 25). In this case, the absence of the E484 mutation makes the Delta variant significantly less resistant to neutralization after infection and vaccination, providing individuals in these circumstances with a stronger protection. Nevertheless, it must be considered that the increased spread of certain viral variants may be not only due to their binding affinity to ACE-2 or capacity to escape from the immune system, but also to their ability to replicate faster than the original virus.

**Figure 4:**
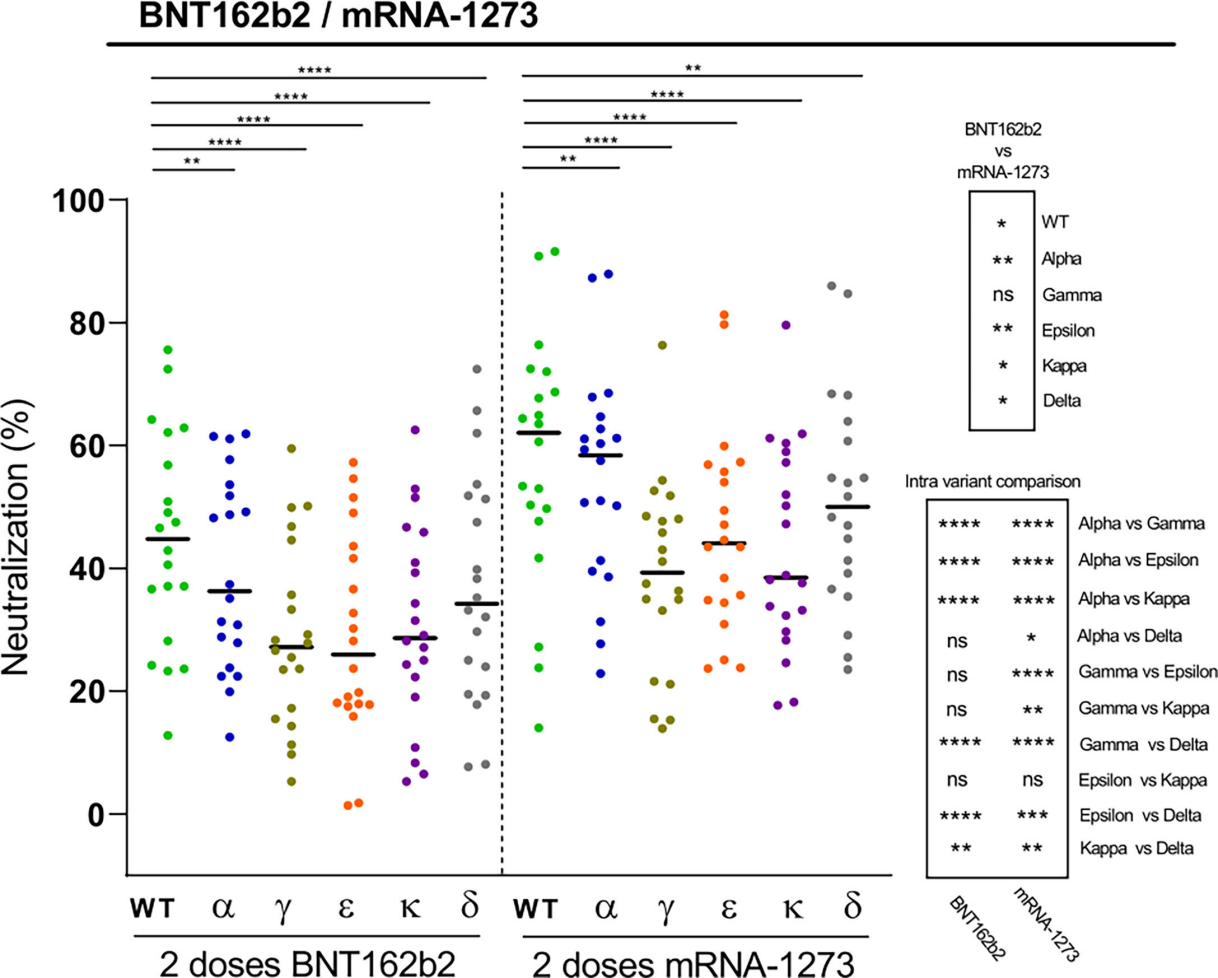
Neutralization of SARS-CoV-2 variants by plasma from BNT162b2 vaccine recipients. Comparative neutralizing capacity by plasma from naïve BNT162b2 (n=20) and mRNA-1273 (n=20) recipients of 2 doses. Plasma dilution used, 1:400. Dots represent the mean of two independent determinations from each individual serum sample analyzed, and black bars indicate median % of neutralization for each group. Statistical differences were determined by Mann-Whitney (for two independent samples) or Wilcoxon (for two relating samples) tests. Statistical significance: *P < 0.05, **P < 0.01, ***P < 0.001, ****P < 0.0001, ns, non-significant. Friedman test *P*-values: 2 doses BNT162b2 = 1.09·10^12^, and 2 doses mRNA-1273 = 2.06·10^-13^.

We finally investigated the neutralization capacity elicited by two doses of the Pfizer-BioNTech (BNT162b2) vaccine against the different RBD variants in naïve individuals. Again, mutations introduced in the RBD variants led to significant reductions in the plasma neutralization potency, with in this case the Gamma, Kappa, and also the Epsilon variants displaying the greatest potential for immune escape, followed by the Delta variant (**Figure 4**). The data also show substantial variations in the magnitude of the responses, with a few cases of BNT162b2 vaccinated individuals with modest RBD-specific neutralizing antibody levels against some variants, suggesting that these subjects may benefit from a third vaccine dose. This finding is of particular relevance, as this may be also the case for individuals with a more fragile immune system such as the elderly population or immunocompromised patients, including organ transplant recipients and patients with cancer (26, 27). We have previously shown that the mRNA-1273 vaccine elicited higher antibody levels against S antigens than the BNT162b2 vaccine (20). Accordingly, when comparing the neutralizing responses induced by BNT162b2 and mRNA-1273 vaccines in plasmas collected at similar time points after the second dose, we observed that the mRNA-1273 vaccine induced higher levels of neutralizing antibodies against all tested variants than the BNT162b2 vaccine (**Figure 4**).

One of the limitations of this study includes the relatively small sample size. However, it must be noted that this study is of exploratory nature and descriptive. Moreover, the sample size was sufficient to reveal differences in the levels of neutralizing antibodies against some of the RBD variants compared with the WT RBD in both, naturally infected and vaccinated individuals. Another limitation of this study is that we do not provide data about the T cell responses in our cohort of health care workers. This remains a relevant area of future study. Finally, we cannot exclude that differences in the timing of sample collection after vaccination may affect, in some cases, the variations observed in the levels of RBD-specific neutralizing antibodies between individuals within the same group. Yet, there are other factors intrinsic to the individuals that may also contribute to the heterogeneity of responses, including age, comorbidities, or smoking habits, as we have previously reported (21).

Altogether, in this study we demonstrate the induction of differentially impaired neutralizing antibody responses against five RBD variants, in both convalescent and mRNA-vaccinated healthcare personnel, with variants carrying E484K/Q displaying the highest neutralizing antibody resistance. We conclude that neutralizing antibody levels largely depend on prior SARS-CoV-2 exposure, number of vaccine doses, and brand of vaccine. In addition, the wide heterogeneity in the magnitude of the neutralizing antibody responses, even taking into consideration the relatively homogeneous healthcare worker group examined, highlights the need of tailored vaccination regimens. Thus, our findings should contribute to a better understanding of SARS-CoV-2 vaccine*-*induced immunity, and underline the importance of close monitoring neutralizing antibody responses against emerging immune escape viral variants to inform future vaccination schemes.

## Data Availaility Statement

The raw data supporting the conclusions of this article will be made available by the authors, without undue reservation.

## Ethics Statement

The study was approved by the Ethics Committee at HCB (references HCB/2020/0336 and HCB/2021/0196). The patients/participants provided their written informed consent to participate in this study.

## Author Contributions

PE and AA designed the study, supervised the work, interpreted the results, and wrote the manuscript. PH-L and JP-P performed laboratory work and data analysis. RA, MT, and CD coordinated participant visits and sample and data collection. RA, AG-B, GM, and CD contributed to the design and critical interpretation of the results. GR-O performed and reviewed the statistical analysis. All authors discussed the results and commented on the manuscript. All authors contributed to the article and approved the submitted version.

## Funding

We acknowledge the European Institute of Innovation and Technology (EIT) Health (grant number 20877), supported by the European Institute of Innovation and Technology, a body of the European Union receiving support from the H2020 Research and Innovation Programme, and the Institut de Salut Global de Barcelona (ISGlobal) internal funds, in-kind contributions from Hospital Clínic de Barcelona and the Fundació Privada Daniel Bravo Andreu. We also acknowledge support from the Spanish Ministry of Science and Innovation (grants SAF2017-87688-R and PID2020-116918RB-100 to AA, and RTI2018-094440-B-100 to PE) and State Research Agency through the “Centro de Excelencia Severo Ochoa 2019-2023” Program (CEX2018-00080-S), and support from the Generalitat de Catalunya though the CERCA Program.

## Conflict of Interest

The authors declare that the research was conducted in the absence of any commercial or financial relationships that could be construed as a potential conflict of interest.

## Publisher’s Note

All claims expressed in this article are solely those of the authors and do not necessarily represent those of their affiliated organizations, or those of the publisher, the editors and the reviewers. Any product that may be evaluated in this article, or claim that may be made by its manufacturer, is not guaranteed or endorsed by the publisher.

## Abbreviations

ACE2, Angiotensin-converting enzyme 2; COVID-19, Coronavirus disease of 2019; MFI, Mean fluorescence intensity; mRNA, Messenger RNA; ns, non-significant; RBD, Receptor-binding domain; SARS-CoV-2, Severe acute respiratory syndrome coronavirus-2; S protein, Spike protein.
