# Supplementary material for: Decreased and Heterogeneous Neutralizing Antibody Responses Against RBD of SARS-CoV-2 Variants After mRNA Vaccination
Source: Front Immunol. 2022 Apr 6;13:816389. doi: 10.3389/fimmu.2022.816389 (PMC9019072; doi:10.3389/fimmu.2022.816389)
Supplement: Supplementary file 1 [file Table_1.docx]

**Supplementary Table 1. Characteristics of study participants.**

| **VACCINATION** | | **DOSES** | **EXPOSURE** | **WITH SIMPTOMS** |
| --- | --- | --- | --- | --- |
| No vaccinated (36) | | *NA* | pre-exposed (36) | 29 |
|  |  |  |  |  |
| Vaccinated (67) | mRNA-1273  (Moderna Biotech)  (47) | 1 dose (27) | naïve (12) | *NA* |
|  |  |  | pre-exposed (15) | 14 |
|  |  | 2 doses (20) | naïve (20) | *NA* |
|  |  |  |  |  |
|  | BNT162b2 (Pfizer-BioNTech)  (20) | 2 doses (20) | naïve (20) | *NA* |

*NA,* Not applicable.
