# Supplementary material for: Decreased and Heterogeneous Neutralizing Antibody Responses Against RBD of SARS-CoV-2 Variants After mRNA Vaccination
Source: Front Immunol. 2022 Apr 6;13:816389. doi: 10.3389/fimmu.2022.816389 (PMC9019072; doi:10.3389/fimmu.2022.816389)
Supplement: Supplementary file 2 [file Table_2.docx]

**Supplementary Table 2. Oligonucleotides employed for the generation of RBD-mFc variants.**

| **Oligo name** | **Sequence (5’-3’)** | **Use** |
| --- | --- | --- |
| EcoRICoV2RBD | GAATTCATGAAGACCATCATCGCCCTGAGC | SOE external primers |
| BglIISARSCoV2RBD | AGATCTCTTTTTGGGCCCACAAACTGTAGC |  |
| N501YCoV2RBD-Rev | ATACCCCACTCCGTATGTTGGTTGAAAGC | Introduction N501Y mutation |
| N501YCoV2RBD-For | GGCTTTCAACCAACATACGGAGTGGGGTAT |  |
| E484KCoV2RBD-Rev | AGCAATTAAATCCCTTTACTCCGTTACATGG | Introduction E484K mutation |
| E484KCoV2RBD-For | CCATGTAACGGAGTAAAGGGATTTAATTGCT |  |
| K417TCoV2RBD-Rev | GTAATCCGCTATGGTCCCAGTCTGACCTGG | Introduction K417T mutation |
| K417TCoV2RBD-For | CCAGGTCAGACTGGGACCATAGCGGATTAC |  |
| L452RCoV2RBD-Rev | AAACAGGCGATACCGGTAATTATAATTACC | Introduction L452R mutation |
| L452RCoV2RBD-For | GGTAATTATAATTACCGGTATCGCCTGTTT |  |
| E484QCoV2 RBD-Rev | AGCAATTAAATCCCTGTACTCCGTTACATGG | Introduction E484Q mutation |
| E484QCoV2RBD-For | CCATGTAACGGAGTACAGGGATTTAATTGCT |  |
| T478KCoV2RBD-Rev | TCCGTTACATGGCTTACTGCCTGCTTG | Introduction T478K mutation |
| T478KCoV2RBD-For | CAAGCAGGCAGTAAGCCATGTAACGGA |  |
